# Activation of ERK1/2 by MOS and TPL2 leads to dasatinib resistance in chronic myeloid leukaemia cells

**DOI:** 10.1111/cpr.13420

**Published:** 2023-02-27

**Authors:** Masanobu Tsubaki, Tomoya Takeda, Yuuichi Koumoto, Takehiro Usami, Takuya Matsuda, Shiori Seki, Kazuko Sakai, Kazuto Nishio, Shozo Nishida

**Affiliations:** ^1^ Division of Pharmacotherapy Kindai University School of Pharmacy Higashi‐Osaka Osaka Japan; ^2^ Department of Genome Biology Kindai University School of Medicine Osakasayama Osaka Japan

## Abstract

The development of BCR::ABL1 tyrosine kinase inhibitors (TKIs), such as dasatinib, has dramatically improved survival in cases of chronic myeloid leukaemia (CML). However, the development of resistance to BCR::ABL1 TKIs is a clinical problem. BCR::ABL1 TKI resistance is known to have BCR::ABL1‐dependent or BCR::ABL1‐independent mechanisms, but the mechanism of BCR::ABL1 independence is not well understood. In the present study, we investigated the mechanism of BCR::ABL1‐independent dasatinib resistance. The expression and activation level of genes or proteins were evaluated using array CGH, real time PCR, or western blot analysis. Gene expression was modulated using siRNA‐mediated knockdown. Cell survival was assessed by using trypan blue dye method. We found that dasatinib‐resistant K562/DR and KU812/DR cells did not harbour a BCR::ABL1 mutation but had elevated expression and/or activation of MOS, TPL2 and ERK1/2. In addition, MOS siRNA, TPL2 siRNA and trametinib resensitized dasatinib‐resistant cells to dasatinib. Moreover, expression levels of MOS in dasatinib non‐responder patients with CML were higher than those in dasatinib responders, and the expression of TPL2 tended to increase in dasatinib non‐responder patients compared with that in responder patients. Our results indicate that activation of ERK1/2 by elevated MOS and TPL2 expression is involved in dasatinib resistance, and inhibition of these proteins overcomes dasatinib resistance. Therefore, MOS, TPL2 and ERK1/2 inhibitors may be therapeutically useful for treating BCR::ABL1‐independent dasatinib‐resistant CML.

## Graphical abstract

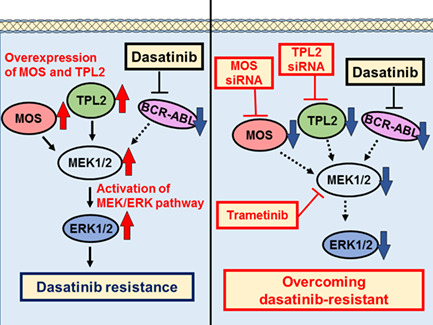

## 1 INTRODUCTION

Chronic myeloid leukaemia (CML) is a myeloproliferative tumour of haematopoietic stem cells that affects 1–2 in 100,000 people, accounting for 15% of all adult leukaemia cases. The Philadelphia chromosome, a chimeric chromosome resulting from a reciprocal translocation of the *Abelson murine viral homologue 1* (*ABL1*) gene on chromosome 9 and the *breakpoint cluster region* (*BCR*) gene on chromosome 22, has been implicated as a major factor in the development of CML.^1^ The Philadelphia chromosome generates BCR::ABL1 protein, which is positive in approximately 95% of CML patients.^2^ BCR::ABL1 protein constitutively activates, Janus kinase (JAK)/signal transducer and activator of transcription (STAT), phosphoinositide 3‐kinase (PI3K)/Akt, and mitogen‐activated protein kinase kinase 1/2 (MEK1/2)/extracellular signal‐regulated kinase 1/2 (ERK1/2) pathways, which are involved in CML cell proliferation and survival.^2^ BCR::ABL1‐dependent activation of Src kinase also contributes to the pathogenesis of CML.^3^ BCR::ABL1 tyrosine kinase inhibitors (TKIs) such as imatinib, nilotinib and dasatinib are currently the first‐line agents for the treatment of CML.^4^ Compared to conventional interferon therapy, these inhibitors have markedly improved the 5‐year survival rate of chronic phase CML patients to more than 90%.^5^ However, approximately 30% of patients who receive BCR::ABL1 TKIs develop resistance or intolerance, which is a major clinical problem.^6^

A cause of BCR::ABL1 TKI resistance is point mutations in the kinase domain within the BCR::ABL1 protein, which interfere with the binding of BCR::ABL1 TKIs to the BCR::ABL1 protein, thereby reducing the sensitivity of the drug.^7^ Currently, there are more than 1000 reported point mutations in the kinase domain of *BCR::ABL1*. Dasatinib is 300 times more potent than imatinib and is effective against most of these mutations.^8^ However, dasatinib has been reported to be ineffective against the T315I mutation, and this mutation accounts for approximately 24% of dasatinib‐resistant patients.^9^ Third‐generation BCR::ABL1 TKIs, ponatinib and rebastinib, have been developed to treat T315I mutations.^10^ This has improved the prognosis in patients with resistant mutations in the *BCR::ABL1* kinase domain. However, approximately 50% of patients who are resistant or intolerant to dasatinib do not have point mutations.^9^ In addition to point mutations in the kinase domain within the BCR::ABL1 protein, other factors that may cause CML to become resistant to dasatinib include activation of the Gab2 or Akt pathway, reduction of FOXO1 or PTEN expression, and activation of Fyn or Lyn kinase.^11^, ^12^, ^13^, ^14^ However, the mechanism of BCR::ABL1‐independent dasatinib resistance remains unclear.

In this study, we established dasatinib‐resistant cell lines using *BCR::ABL1*‐positive human CML cell lines K562 and KU812 and investigated the mechanism of dasatinib‐acquired resistance.

## 2 MATERIALS AND METHODS

### 2.1 Cell lines

K562 cells were procured from The Japanese Collection of Research Bioresources Cell Bank, recently authenticated by DNA STR profiling (Promega). These cell lines were used to establish dasatinib‐resistant lines (K562/DR), as previously described.^15^, ^16^ Mycoplasma‐free status was confirmed every 6 months (TaKaRa PCR Mycoplasma Detection Set, Takara Biomedical).

### 2.2 Cell viability assay

Cell survival and proliferation by treatment with various reagents was measured using the trypan blue dye staining assay, as previously described.^17^, ^18^

### 2.3 Array comparative genomic hybridization

Genomic DNA was analysed using OncoScan FFPE Assay (Affymetrix) according to the manufacturer's protocol, as previously described.^19^

### 2.4 Statistical analysis

All data are presented as the mean ± standard deviation of several independent experiments, and the data were analysed using analysis of variance with Dunnet test. Significant differences were determined at *p* < 0.05.

Other materials and methods is referred to the supplementary materials and methods.

## 3 RESULTS

### 3.1 Effect of dasatinib and other BCR::ABL1 TKIs on dasatinib‐resistant cells

**FIGURE 1:**
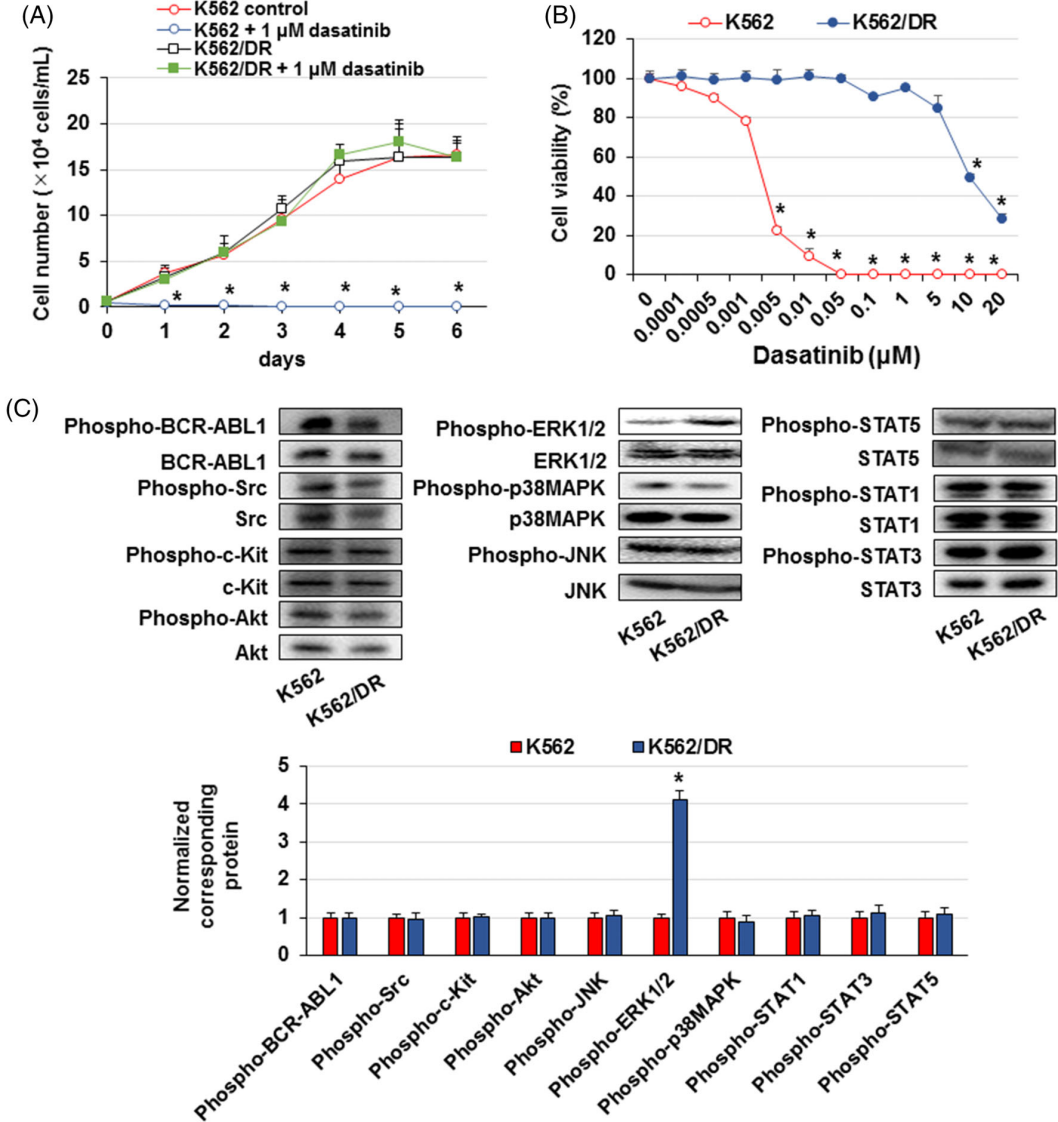
Increased activation of ERK1/2 in K562/DR cells. (A) K562/DR and K562 cells were treated with 1 μM dasatinib. After 1–6 days, the number of surviving/dead cells was examined by trypan blue staining. These results are the average of five independent experiments. *p < 0.01 versus untreated K562 cells. (B) Cell survival of K562/DR and K562 cells after treatment with various concentrations of dasatinib for 72 h. These results are the average of five independent experiments. *p < 0.01 versus untreated K562 cells. (C) A Cell lysates were analysed by western blotting. Phosphorylated proteins were analysed by densitometry and were standardized to the corresponding total proteins.

We examined whether dasatinib suppressed K562 and K562/DR cell proliferation. Treatment with 1 μM dasatinib inhibited cell proliferation in K562 cells but did not affect K562/DR cells (Figure 1A). In addition, the survival of K562 and K562/DR cells was investigated using IC50 values for dasatinib and other BCR::ABL1 TKIs, such as imatinib, nilotinib, bafetinib, ponatinib, rebastinib, GNF‐2 and GNF‐5. We found that K562/DR cells showed a markedly higher IC50 value than K562 cells (Figure 1B; Figure S1A,B).

We also examined the *ABL1* gene mutation related to BCR::ABL1 TKIs resistance in K562/DR cells. The NGS analysis did not reveal any mutations in the *ABL1* gene in K562/DR cells (Figure S1C).

### 3.2 Constitutive activation of ERK1/2 in K562/DR cells did not affect other targeted signalling molecules by BCR::ABL1 TKIs

We first examined the activation of signalling molecules directly inhibited by dasatinib and their downstream components to analyse the factors that contribute to the acquisition of dasatinib resistance. We found that the activation levels of ERK1/2 were higher in K562/DR cells than in K562 cells, but activation of BCR‐ABL1, Src, c‐Kit, Akt, JNK, p38MAPK, STAT1, STAT3 and STAT5 did not differ between K562 and K562/DR cells (Figure 1C).

**FIGURE 2:**
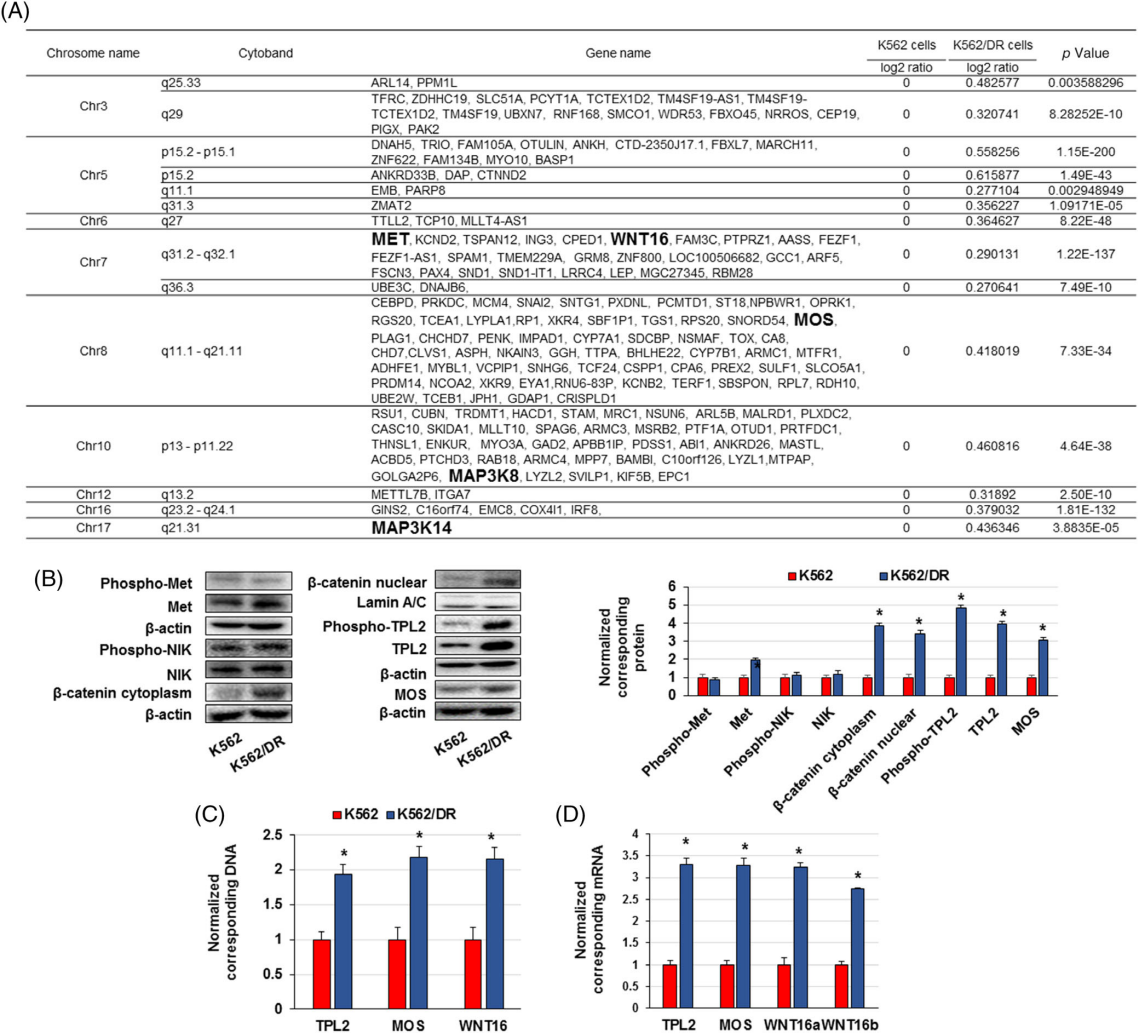
Increased expression and/or activation of TPL2 and MOS in K562/DR cells. (A) Elevated copy number variation in K562/DR cells compared to K562 cells; values are in log2 ratios. (B) Cell lysates were analysed by western blotting. Proteins were analysed by densitometry and were standardized to β‐actin or Lamin A/C. (C) Genomic DNA and (D) RNA were extracted, and TPL2, MOS and WNT16 levels were examined by real time PCR. The results were standardized using GAPDH values and then expressed as a test: Control ratio. These results are the average of five independent experiments. *p < 0.01 versus untreated K562 cells.

Next, we examined the copy number variation (CNV) in K562 and K562/DR cells to analyse the mechanism of ERK1/2 activation and other genes involved in dasatinib resistance. These examinations showed a large number of genomic DNA that were amplified only in K562/DR cells, but not in K562 cells. Among them, we focused on five genes that were amplified in K562/DR cells: *MET*, *WNT16*, *MOS*, *mitogen‐activated protein kinase kinase kinase 8* (*MAP3K8*; also known as *TPL2*), and *MAP3K14* (also known as *NF‐κB inducing kinase* [*NIK*]) (Figure 2A). MET is a receptor tyrosine kinase that accelerates cell survival, growth and resistance to molecular therapies targeting BCR::ABL1, MEK, BRAF and epithelial growth factor receptor.^15^, ^20^ WNT16 is a member of the WNT family and is concerned in tumour cell growth and survival via activation of β‐catenin.^21^ MOS and TPL2 are upstream signal molecules of ERK1/2, and TPL2 overexpression is involved in resistance to imatinib in CD34+ CML cells.^22^, ^23^ In addition, we confirmed that MET, cytoplasmic β‐catenin, nuclear β‐catenin, phosphorylated TPL2, TPL2 and MOS were higher in K562/DR cells than in K562 cells, but there was no change in NIK expression between K562 and K562/DR cells (Figure 2B). We also found that the amplification levels of genomic DNA and mRNA of TPL2, MOS and WNT16 in K562/DR cells were higher than those in K562 cells (Figure 2C,D). Thus, TPL2 and MOS may activate ERK1/2, and WNT16 may activate β‐catenin, leading to dasatinib resistance in CML cells.

### 3.3 Activation of ERK1/2 by MOS and TPL2 is involved in dasatinib resistance, but not WNT16

**FIGURE 3:**
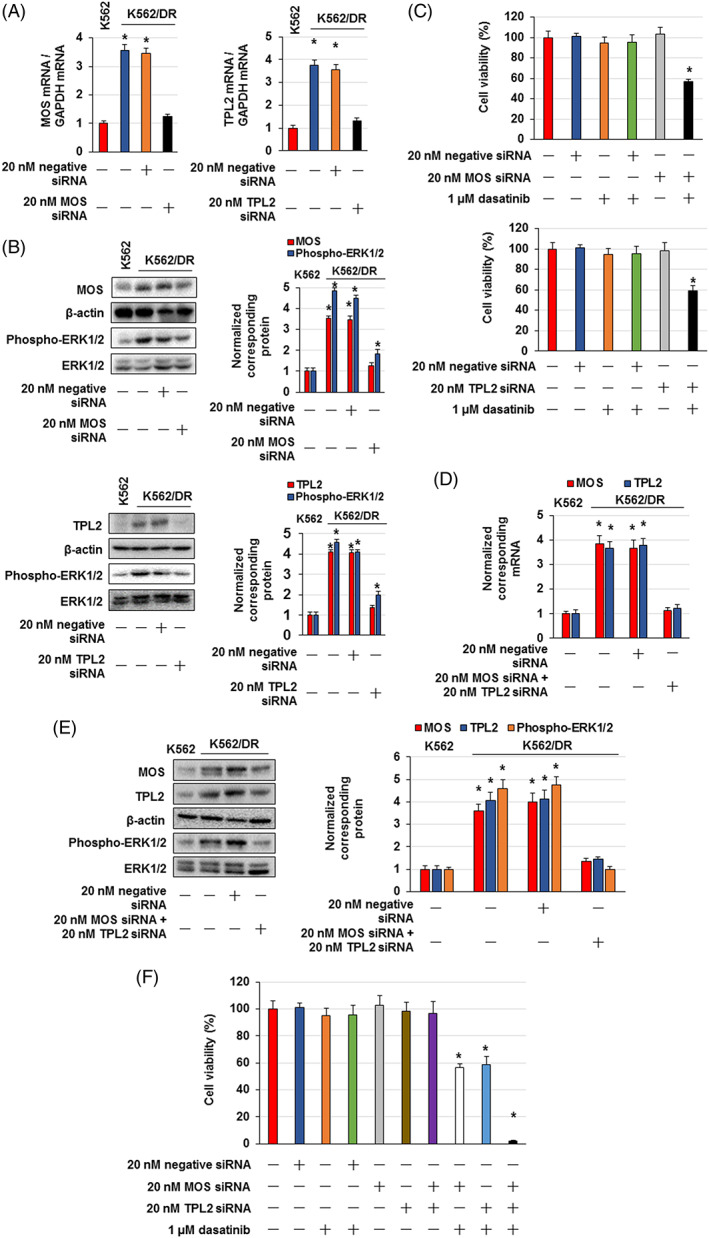
Inhibition of MOS, TPL2 and ERK1/2 overcame dasatinib resistance in K562/DR cells. (A) K562/DR cells were administrated with siRNA of MOS, TPL2, or a negative control for 1 day, and RNA were extracted. TPL2 and MOS levels were examined by real time PCR. The results were standardized using GAPDH values and then expressed as a test: Control ratio. These results are the average of five independent experiments. *p < 0.01 versus untreated K562 cells. (B) Cell lysates were analysed by western blotting. Total or phosphorylated proteins were analysed by densitometry and were standardized to β‐actin or ERK1/2. (C) K562/DR cells were administrated with the demonstrated concentrations of MOS siRNA, TPL2 siRNA, or dasatinib. After incubation for 72 h, the number of surviving/dead cells was determined by trypan blue staining. These results are the average of five independent experiments. *p < 0.01 versus untreated K562/DR cells. (D–F) K562/DR cells were treated with MOS siRNA and TPL2 siRNA or dasatinib. (D) RNA was extracted, and MOS and TPL2 levels were examined by real time PCR. The results were standardized using GAPDH values and then expressed as a test: Control ratio. These results are the average of five independent experiments. *p < 0.01 versus untreated K562 cells. (E) Cell lysates were analysed by western blotting. Total or phosphorylated proteins were analysed by densitometry and were standardized to β‐actin or ERK1/2. (F) K562/DR cells were administrated with 20 nM MOS siRNA, 20 nM TPL2 siRNA or 1 μM dasatinib. After incubation for 72 h, the number of surviving/dead cells was determined by trypan blue staining. These results are the average of five independent experiments. *p < 0.01 versus untreated K562/DR cells.

**FIGURE 4:**
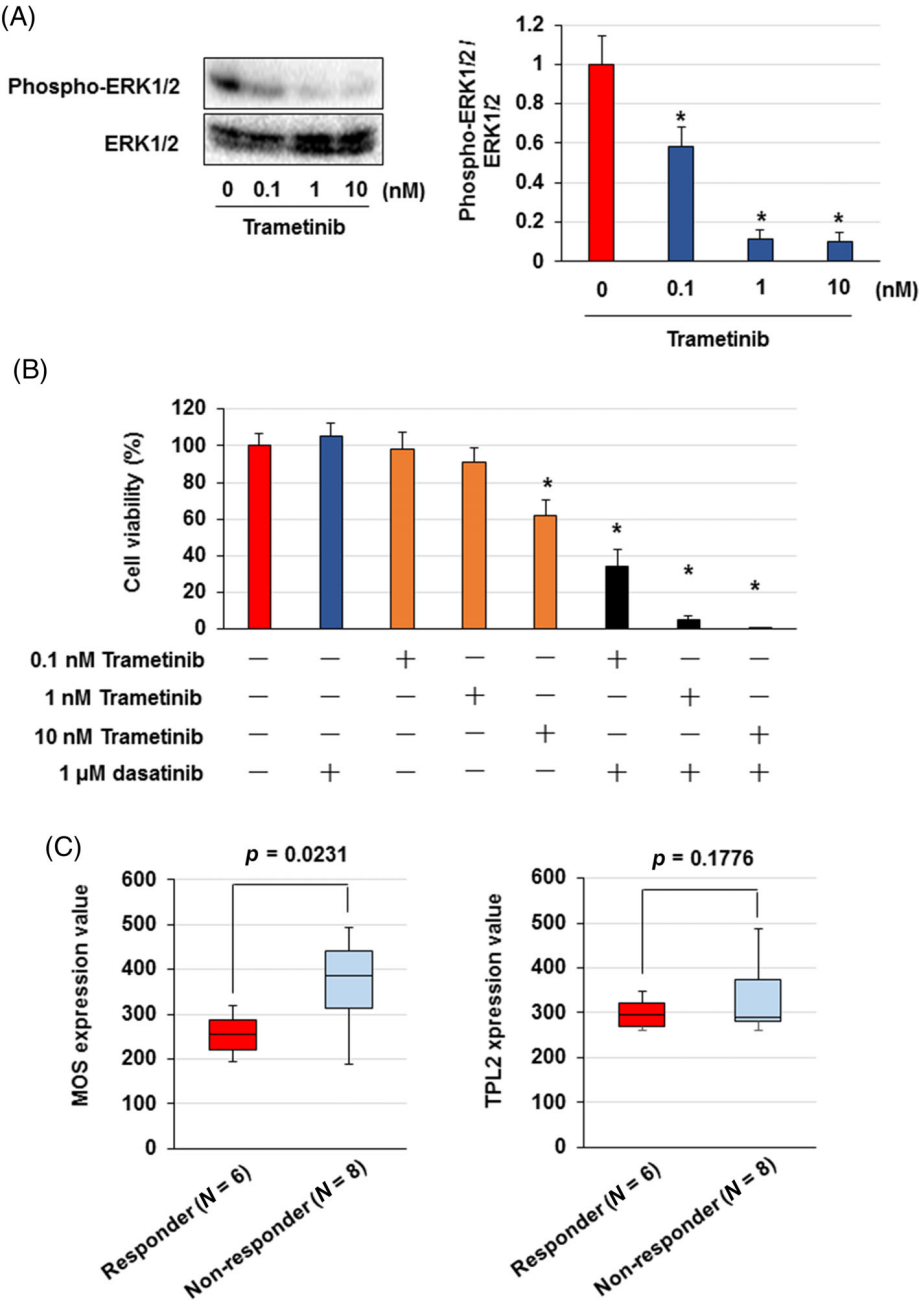
Trametinib resensitized the K562/DR cells to dasatinib. (A, B) K562/DR cells were administrated with trametinib for 72 hr. (A) Cell lysates were analysed by western blotting. Phosphorylated ERK1/2 were analysed by densitometry and were standardized to ERK1/2. (B) K562/DR cells were administrated with 0.1, 1 and 10 nM trametinib or 1 μM dasatinib. After incubation for 72 h, the number of survival/dead cells was determined by trypan blue staining. These results are the average of five independent experiments. *p < 0.01 versus untreated K562/DR cells. (C) The expression levels of MOS and TPL2 in dasatinib non‐responder patients was analysed using the GSE33224 datasets.

We observed that MOS, TPL2 and WNT16 knockdown by siRNA resensitized K562/DR cells to dasatinib. MOS or TPL2 siRNAs alone suppressed MOS and TPL2 expression and inhibited ERK1/2 activation in K562/DR cells, but the activation level of ERK1/2 was higher than that of K562 cells (Figure 3A,B). In addition, MOS or TPL2 siRNAs alone elevated the sensitivity of K562/DR cells to dasatinib (Figure 3C). WNT16 siRNA markedly inhibited WNT16 mRNA and nuclear β‐catenin expression in K562/DR cells but did not affect the viability of K562/DR cells (Figure S2). Combined treatment with MOS and TPL2 siRNAs significantly inhibited ERK1/2 phosphorylation and overcame dasatinib resistance in K562/DR cells (Figure 3D–F). Moreover, trametinib, an MEK1/2 inhibitor, suppressed ERK1/2 phosphorylation and resensitized K562/DR cells to dasatinib in (Figure 4A,B).

We also analysed whether MOS and TPL2 expression were different between dasatinib non‐responder and responder patients using the GSE33224 dataset. We found that the expression levels of MOS in dasatinib non‐responders were higher than those in dasatinib responders (Figure 4C), and the expression level of TPL2 in dasatinib non‐responders tended to increase more than that in dasatinib responders, although the difference was not significant (Figure 4C). Therefore, high expression of MOS and TPL2 was correlated with dasatinib resistance in patients with CML.

To validate these findings, we examined whether the expression of MOS, TPL2, and phosphorylated ERK1/2 contributed to dasatinib resistance in KU812/DR cells. KU812/DR cells, like K562/DR cells, were resistant to various BCR::ABL1 TKIs, did not have BCR‐ABL mutations, and had elevated the expression of MOS, TPL2, and phosphorylated ERK1/2 (Figures S3 and S4A,B). Moreover, trametinib overcame dasatinib resistance in KU812/DR cells (Figure S4C). It has been reported that the activation of NF‐κB promotes TPL2 expression in imatinib‐resistant CML cells.^23^ We also investigated the activation/expression of NF‐κB p65 in dasatinib‐resistant CML cells. There was no change in phosphorylated or total NF‐κB p65 protein levels between parental and resistant cells (Figure S5).

## 4 DISCUSSION

In the present study, we established dasatinib‐resistant K562/DR and KU812/DR cells that were resistant to dasatinib and other BCR::ABL1 TKIs, such as nilotinib, ponatinib, rebastinib and GNF‐5. In addition, K562/DR and KU812/DR cells did not harbour a kinase domain mutation in *BCR::ABL1* correlated with BCR::ABL1 TKI resistance. It has been reported that treatment for BCR::ABL1 mutations is described in therapeutic guidelines, but there is no description of what to do in the absence of BCR::ABL1 mutations.^24^ Our results indicated that the established cells are resistant by a BCR::ABL1‐independent mechanism for which there are few countermeasures that are clinically problematic in CML patients.

Dasatinib inhibits the activation of BCR::ABL1, Src, c‐Kit, and alternative signalling pathways that contribute to BCR::ABL1 TKI resistance, such as PI3K/Akt, JAK/STAT and MEK/ERK pathways.^24^ K562/DR cells showed increased ERK1/2 phosphorylation compared to K562 cells, but there was no change in the activation of BCR::ABL1, Src, c‐Kit, Akt, JNK, p38MAPK, STAT1, STAT3 and STAT5 between K562/DR and K562 cells. We examined the upstream processes of ERK1/2 and other factors involved in dasatinib resistance using CNV analysis and found increased gene expression of *MET*, *WNT16*, *MOS*, *TPL2* (*MAP3K8*), and *NIK* (*MAP3K14*) in K562/DR cells. In addition, the protein expression of MET and β‐catenin, which is a downstream signalling molecule of WNT16, MOS and TPL2, was elevated in K562/DR cells, but NIK expression and levels of phosphorylated MET were unchanged. Moreover, treatment with siRNA of MOS and TPL2, or trametinib resensitized the cells to dasatinib through inhibition of ERK1/2 activation in K562/DR cells. However, despite decreasing the nuclear localization of β‐catenin with WNT16 siRNA, dasatinib resistance was not observed. Furthermore, we found that dasatinib non‐responder patients had elevated MOS expression and a trend towards increased TPL2 expression compared to dasatinib responders. It has been indicated that inhibition of MOS by treatment with shRNA suppresses ERK phosphorylation and enhances the induction of apoptosis by ABT‐869, a multikinase inhibitor, in human acute myeloid leukaemia cells.^25^ Moreover, overexpression of MOS by p110 CUX1 induced ERK1/2 phosphorylation, cell proliferation and tumorigenesis in pancreatic cancer.^26^ It has also been reported that high expression of MOS is associated with poor prognosis in patients with lung adenocarcinoma than in patients with low expression.^27^ In addition, TPL2 overexpression was associated with imatinib resistance in patients with CD34‐positive CML cells.^23^ Elevated expression of TPL2 in BRAF V600E‐mutated melanoma cells is known to confer resistance to BRAF inhibitors, and TPL2 expression is higher in patients with relapse after BRAF inhibitor therapy.^28^ These findings indicate that overexpression of MOS and TPL2 is involved in dasatinib resistance in CML cells.

We showed that the activation of ERK1/2 via MOS and TPL2 overexpression is implicated in dasatinib resistance in CML, but our study has some limitations. First, the GSE dataset was used in this study to examine MOS and TPL2 expression in dasatinib‐non‐responder patients with CML, but the sample size was small. More samples are needed to determine the extent to which the overexpression of MOS and TPL2 contributes to dasatinib resistance in patients with CML. Second, we did not use CML cells from patients with dasatinib‐resistant CML overexpressing MOS and TPL2 to confirm whether the abrogation of MOS, TPL2 and ERK1/2 overcomes dasatinib resistance. This is important to elucidate the mechanism of dasatinib resistance in CML and to establish a therapeutic strategy, which needs to be clarified in a subsequent study.

**FIGURE 5:**
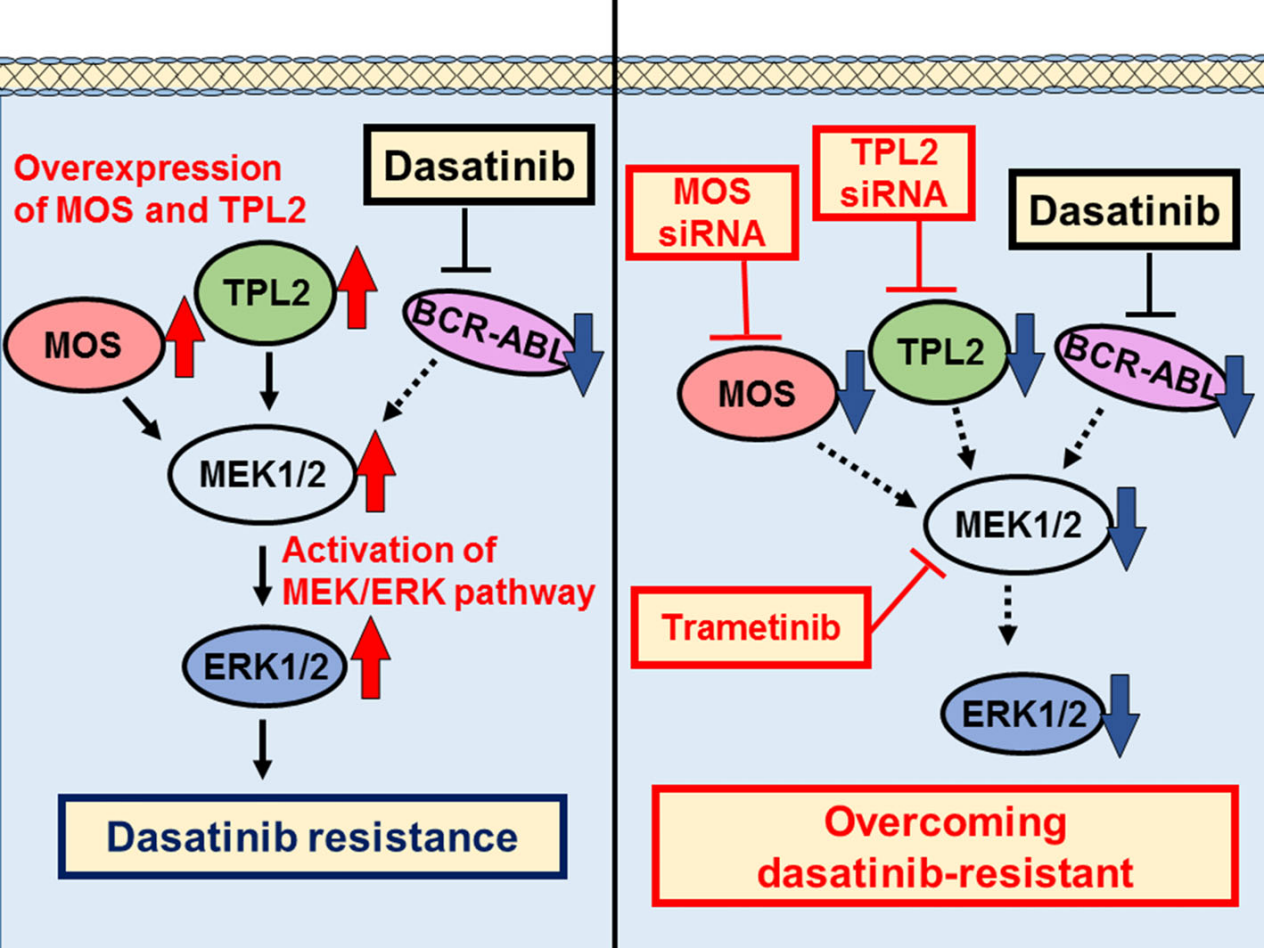
Schematic summary of dasatinib resistance mechanism in chronic myeloid leukaemia (CML) cells. Activation of ERK1/2 by the overexpression of MOS and TPL2 was associated with BCR::ABL1‐independent dasatinib resistance in CML cells. Moreover, the inhibition of MOS and TPL2, ERK1/2 activation by siRNAs targeting *MOS* and *TPL2*, or trametinib treatment overcame dasatinib resistance.

In conclusion, we demonstrated that overexpression of MOS and TPL2 is associated with BCR::ABL1‐independent dasatinib resistance in CML cells and that inhibition of MOS and TPL2 or ERK1/2 activation by MEK1/2 inhibitor overcomes dasatinib resistance (Figure 5). These findings suggest that a combination of MOS, TPL2 or MEK inhibitor and dasatinib may be a useful strategy for the treatment of BCR::ABL1‐independent dasatinib‐resistant CML.

## AUTHOR CONTRIBUTIONS

**Shozo Nishida**: Conceptualization and design. **Masanobu Tsubaki**, **Tomoya Takeda**, **Yuuichi Koumoto**, **Takehiro Usami**, **Takuya Matsuda**, **Shiori Seki**, **Kazuko Sakai** and **Kazuto Nishio**: Acquisition of data; analysis and interpretation of data; experiment performance. **Masanobu Tsubaki**: Manuscript writing. All authors read and approved the final manuscript.

## FUNDING INFORMATION

This work was supported in part by a Grant‐in‐Aid for Scientific Research (C) (Grant numbers 20K07145 and 20K07168) from the Japan Society for the Promotion of Science (JSPS).

## CONFLICT OF INTEREST STATEMENT

The authors declare no conflict of interest.

## Supporting information


**Data S1:** Supporting Information

**Figure S1.** BCR::ABL1 TKIs treatment did not increase cell death in K562/DR cells. The effect of BCR::ABL1 TKIs on cell growth/survival was decided using the trypan blue staining assay. (A) Cell survival of K562/DR and K562 cells after treatment with various concentrations of imatinib, nilotinib, bafetinib, ponatinib, rebastinib, GNF‐2 and GNF‐5 for 72 h; These results are the average of five independent experiments. *p < 0.01 versus untreated K562 cells. (B) The IC50 was evaluated by using a logistic curve for the data. (C) BCR::ABL1 mutations in K562/DR cells was examined by NGS.

**Figure S2.** Effect of WNT16 siRNA on dasatinib resistance of K562/DR cells. (A) K562/DR cells were administrated with siRNA of WNT16 or a negative control for 1 day, and RNA were extracted. WNT16 levels were examined by real time PCR. The results were standardized using GAPDH values and then expressed as a test: control ratio. These results are the average of five independent experiments. *p < 0.01 versus untreated K562 cells. (B) Cell lysates were analysed by western blotting. β‐catenin was analysed by densitometry and were standardized to Lamin A/C. (C) K562/DR cells were administrated with the demonstrated concentrations of WNT16 siRNA or dasatinib. After incubation for 72 h, the number of surviving/dead cells was determined by trypan blue staining. These results are the average of five independent experiments. *p < 0.01 versus untreated K562/DR cells.

**Figure S3.** Dasatinib and other BCR::ABL1 TKIs treatment did not increase cell death in KU812/DR cells. (A) Survival of KU812/DR and KU812 cells after treatment to various concentrations of dasatinib, nilotinib, ponatinib, rebastinib and GNF‐5 for 72 h; These results are the average of five independent experiments. *p < 0.01 versus untreated KU812 cells (evaluated by Dunnett's test). (B) The IC50 was evaluated by using a logistic curve for the data. (C) BCR::ABL1 mutations in KU812/DR cells were examined by NGS.

**Figure S4.** Elevated expression of MOS, TPL2, and ERK1/2 contributed to dasatinib resistance in KU812/DR cells. (A) Cell lysates were analysed by western blotting. Proteins were analysed by densitometry and were standardized to β‐actin or ERK1/2. (B) KU812/DR cells were administrated with trametinib for 72 h. Cell lysates were analysed by western blotting. Phosphorylated ERK1/2 were analysed by densitometry and were standardized to ERK1/2. (C) KU812/DR cells were administrated with 0.1, 1, and 10 nM trametinib or 300 nM dasatinib. After incubation for 72 h, the number of surviving/dead cells was determined by trypan blue staining. These results are the average of five independent experiments. *p < 0.01 versus untreated KU812/DR cells (evaluated by Dunnett's test).

**Figure S5.** Expression of phosphorylated and total NF‐κB p65 on K562, K562/DR, KU812 and KU812/DR cells. (A) K562 and K562/DR, (B) KU812 and KU812/DR cell lysates were analysed by western blotting. Proteins were analysed by densitometry and were standardized to β‐actin.

## Data Availability

The data that support the findings of this study are available from the corresponding author upon reasonable request.
