# Supplementary material for: Activation of ERK1/2 by MOS and TPL2 leads to dasatinib resistance in chronic myeloid leukaemia cells
Source: Cell Prolif. 2023 Feb 27;56(6):e13420. doi: 10.1111/cpr.13420 (PMC10280141; doi:10.1111/cpr.13420)
Supplement: Supplementary file 1 — Data S1: Supporting Information [file CPR-56-e13420-s002.docx]

**Supplementary materials and methods**

Reagents

Bafetinib, dasatinib, GNF-2, GNF-5, imatinib, nilotinib, ponatinib, and rebastinib were obtained from Selleckchem (Houston, TX, USA). Trametinib was obtained from LC Laboratories (Woburn, MA, USA). All reagents were dissolved in dimethyl sulfoxide.

Cell lines

KU812 cells were procured from The Japanese Collection of Research Bioresources Cell Bank (Osaka, Japan), recently authenticated by DNA STR profiling (Promega, Tokyo, Japan). These cell lines were used to establish dasatinib-resistant lines (KU812/DR), as previously described.^1^ Mycoplasma-free status was confirmed every 6 months (TaKaRa PCR Mycoplasma Detection Set, Takara Biomedical, Ohtsu, Japan).

Mutation analysis by next generation sequencing (NGS)

Genomic DNA was extracted from K562 and K562/DR cells. NGS was conducted on an Illumina HiSeq 2500 employing the SureSelectXT Human protocol (Agilent) according to the manufacturer's instructions (Illumina). Paired-end sequencing with 100 bp read length and 100X average on-target coverage was achieved. Human variants were called using SAMtools and the Genome Analysis Toolkit (GATK-Unified Genotyper).

Quantitative genomic polymerase chain reaction (PCR)

Genomic DNA expression was analyzed employing quantitative real-time PCR using Thermal Cycler Dice Real Time system (Takara Biomedical, Ohtsu, Japan) and SYBR Premix Ex Taq (Takara Biomedical). The PCR settings for GAPDH, MOS, tumor progression locus 2 (TPL2), and wingless-type MMTV integration site family member 16 (WNT16) were 94°C for 2 min, followed by 40 cycles of 94°C for 0.5 min, 50°C for 0.5 min, and 72°C for 0.5 min. The following primers were used: MOS, 5′- CAA ACC GAG CCA CAT GTT CC -3′ (5′-primer) and 5′-TCC CTA GGC TAT TGG ACC CT-3′ (3′-primer); TPL2, 5′-ACT TGC ATC CAC GAC CGA TT-3′ (5′-primer) and 5′-TTC TGG ACT CCG CAC TCA TT-3′ (3′-primer); WNT16, 5′-AGA ATG TCC ACC AAC CCG TA-3′ (5′-primer) and 5′-TGC AGT TCC ATC TCT CGT GT-3′ (3′-primer); and GAPDH, 5′-GTC CGA CAT TTA CAG TGG CC-3′ (5′-primer) and 5′-TGA ATG GAC ACG AGG GTG AG-3′ (3′-primer). For standardization, GAPDH was used as an internal control for each sample; Cycle threshold (Ct) values were archived and the standardized expression of each gene in K562 and K562/DR cells was evaluated using the 2^–∆∆Ct^ method.

Western blotting

Cytoplasmic and nuclear fractions extracted from each cells using the ProteoExtract Subcellular Proteome Extraction Kit (Calbiochem, San Diego, CA, USA) were detected by western blotting, as previously described.^1^ The following primary antibodies were used: anti-phospho-ABL1 antibody (#2865), anti-ABL1 antibody (#2862), anti-phospho-Src antibody (#2105), anti-Src antibody (#2108), anti-phospho-c-Kit antibody (#3073), anti-c-Kit antibody (#3074), anti-phospho-Akt antibody (#9271), anti-Akt antibody (#9272), anti-phospho-JNK antibody (#9251), anti-JNK antibody (#9252), anti-phospho-ERK1/2 antibody (#9101), anti-ERK1/2 antibody (#9102), anti-phospho-p38MAPK antibody (#9211), anti-p38MAPK antibody (#9212), anti-phospho-STAT1 antibody (#9167), anti-STAT1 antibody (#9172), anti-phospho-STAT3 antibody (#9131), anti-STAT3 antibody (#4904), anti-phospho-STAT5 antibody (#9351), anti-STAT5 antibody (#94205), anti-phospho-MET antibody (#3126), anti-MET antibody (#4560), anti-β-catenin antibody (#9252), anti-phospho-TPL2 antibody (#4491), anti-TPL-2 antibody (#71184) (Cell Signaling Technology, Beverly, MA, USA), anti-MOS antibody (PA5-44590) (Invitrogen, Carlsbad, CA, USA), anti-phospho-NIK antibody (sc-12957), anti-NIK antibody (sc-7211), anti-Lamin A/C antibody (sc-7293) (Santa Cruz Biotechnologies, CA, USA), and anti-β-actin antibody (A2228, clone AC-74) (Sigma-Aldrich, St Louis, MO, USA).

RNA interference

The small interfering RNAs (siRNAs) against MOS (HSS106672), TPL2 (HSS102181), WNT16 (HSS122128), and Stealth^TM^ RNAi negative control duplex with low GC construct (negative control) were obtained from Invitrogen. Transfection of siRNA was conducted according to the manufacturer’s protocol, as previously described.^1^

Quantitative real-time (PCR)

Total RNA was extracted using RNAiso (Takara Biomedical), and cDNA was synthesized from the extracted RNA using the PrimeScript First-Strand Synthesis System (Takara Biomedical). The cDNA was analyzed by real-time PCR using Thermal Cycler Dice Real Time System (Takara Biomedical) and SYBR Premix Ex Taq (Takara Biomedical). The PCR settings for amplification of GAPDH, MOS, TPL2, and WNT16 gene were 94 °C for 2 min followed by 40 cycles of 94 °C for 0.5 min, 50 °C for 0.5 min, and 72 °C for 0.5 min. The following primer sequences were used; MOS: 5′- CGG TGT TCC GTG GCC ATA A -3′ (5′-primer) and 5′- GAT GTT GTG AAC GGC CTG CT -3′ (3′-primer), TPL2: 5′- CAG TAA TCA AAA CGA TGA GCG TTC TA -3′ (5′-primer) and 5′- ACG TTA CCA AAT AGA TTC CGA TGT TC -3′ (3′-primer), WNT16-2: 5′- CAG AAA GAT GGA AAG GCA CC -3′ (5′-primer) and 5′- GAG AGA TGG AAC TGC ATG AT -3′ (3′-primer), and GAPDH: 5′-GAC ATC AAG GTG AA-3′ (5′-primer) and 5′-TGT CAT ACC AGG AAA TGA GC-3′ (3′-primer). For standardization, GAPDH was used as an internal control for each sample; Cycle threshold (Ct) values were archived and the standardized expression of each gene in K562 and K562/DR cells was evaluated using the 2^–∆∆Ct^ method.

Gene expression omnibus (GEO) dataset

The gene expression profiling with microarray dataset, accession number GSE33224, was procured from the National Center of Biotechnology Information (NCBI) GEO database (http://www.ncbi.nlm.nih.gov/geo/). MOS and TPL2 genes expression in dasatinib responders or non-responders with CML patients was analyzed.

References

1. Tsubaki M, Takeda T, Kino T, Sakai K, Itoh T, Imano M, et al. Contributions of MET activation to BCR-ABL1 tyrosine kinase inhibitor resistance in chronic myeloid leukemia cells. Oncotarget. 2017;8:38717-30.

Supplementary Figure legends

**Figure S1** BCR::ABL1 TKIs treatment did not increase cell death in K562/DR cells. The effect of BCR::ABL1 TKIs on cell growth/survival was decided using the trypan blue staining assay. (A) Cell survival of K562/DR and K562 cells after treatment with various concentrations of imatinib, nilotinib, bafetinib, ponatinib, rebastinib, GNF-2, and GNF-5 for 72 h; These results are the average of five independent experiments. *p < 0.01 vs. untreated K562 cells. (B) The IC50 was evaluated by using a logistic curve for the data. (C) BCR::ABL1 mutations in K562/DR cells was examined by NGS.

**Figure S2** Effect of WNT16 siRNA on dasatinib resistance of K562/DR cells. (A) K562/DR cells were administrated with siRNA of WNT16 or a negative control for 1 day, and RNA were extracted. WNT16 levels were examined by real time PCR. The results were standardized using GAPDH values and then expressed as a test:control ratio. These results are the average of five independent experiments. *p < 0.01 vs. untreated K562 cells. (B) Cell lysates were analyzed by western blotting. β-catenin was analyzed by densitometry and were standardized to Lamin A/C. (C) K562/DR cells were administrated with the demonstrated concentrations of WNT16 siRNA or dasatinib. After incubation for 72 h, the number of surviving/dead cells was determined by trypan blue staining. These results are the average of five independent experiments. *p < 0.01 vs. untreated K562/DR cells.

**Figure S3** Dasatinib and other BCR::ABL1 TKIs treatment did not increase cell death in KU812/DR cells. (A) Survival of KU812/DR and KU812 cells after treatment to various concentrations of dasatinib, nilotinib, ponatinib, rebastinib, and GNF-5 for 72 h; These results are the average of five independent experiments. *p < 0.01 vs. untreated KU812 cells (evaluated by Dunnett's test). (B) The IC50 was evaluated by using a logistic curve for the data. (C) BCR::ABL1 mutations in KU812/DR cells were examined by NGS.

**Figure S4** Elevated expression of MOS, TPL2, and ERK1/2 contributed to dasatinib resistance in KU812/DR cells. (A) Cell lysates were analyzed by western blotting. Proteins were analyzed by densitometry and were standardized to β-actin or ERK1/2. (B) KU812/DR cells were administrated with trametinib for 72 hr. Cell lysates were analyzed by western blotting. Phosphorylated ERK1/2 were analyzed by densitometry and were standardized to ERK1/2. (C) KU812/DR cells were administrated with 0.1, 1, and 10 nM trametinib or 300 nM dasatinib. After incubation for 72 h, the number of surviving/dead cells was determined by trypan blue staining. These results are the average of five independent experiments. *p < 0.01 vs. untreated KU812/DR cells (evaluated by Dunnett's test).

**Figure S5** Expression of phosphorylated and total NF-κB p65 on K562, K562/DR, KU812, and KU812/DR cells. (A, B) (A) K562 and K562/DR, (B) KU812 and KU812/DR cell lysates were analyzed by western blotting. Proteins were analyzed by densitometry and were standardized to β-actin.
