# Supplementary figures and images for: Activation of ERK1/2 by MOS and TPL2 leads to dasatinib resistance in chronic myeloid leukaemia cells
Source: Cell Prolif. 2023 Feb 27;56(6):e13420. doi: 10.1111/cpr.13420 (PMC10280141; doi:10.1111/cpr.13420)

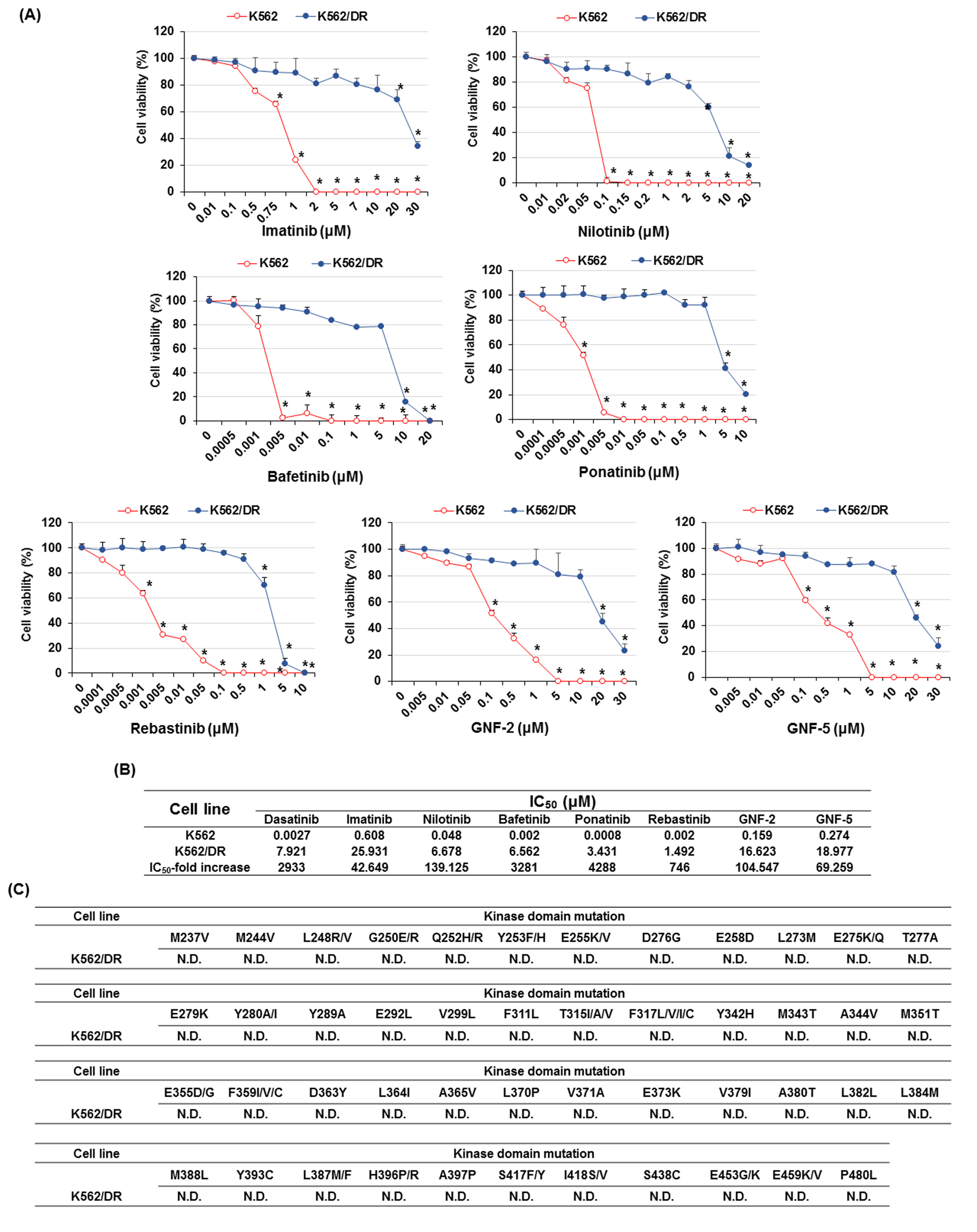

Supplement: Supplementary file 2 — Figure S1. BCR::ABL1 TKIs treatment did not increase cell death in K562/DR cells. The effect of BCR::ABL1 TKIs on cell growth/survival was decided using the trypan blue staining assay. (A) Cell survival of K562/DR and K562 cells after treatment with various concentrations of imatinib, nilotinib, bafetinib, ponatinib, rebastinib, GNF‐2 and GNF‐5 for 72 h; These results are the average of five independent experiments. *p < 0.01 versus untreated K562 cells. (B) The IC50 was evaluated by using a logistic curve for the data. (C) BCR::ABL1 mutations in K562/DR cells was examined by NGS. [file CPR-56-e13420-s006.tif]

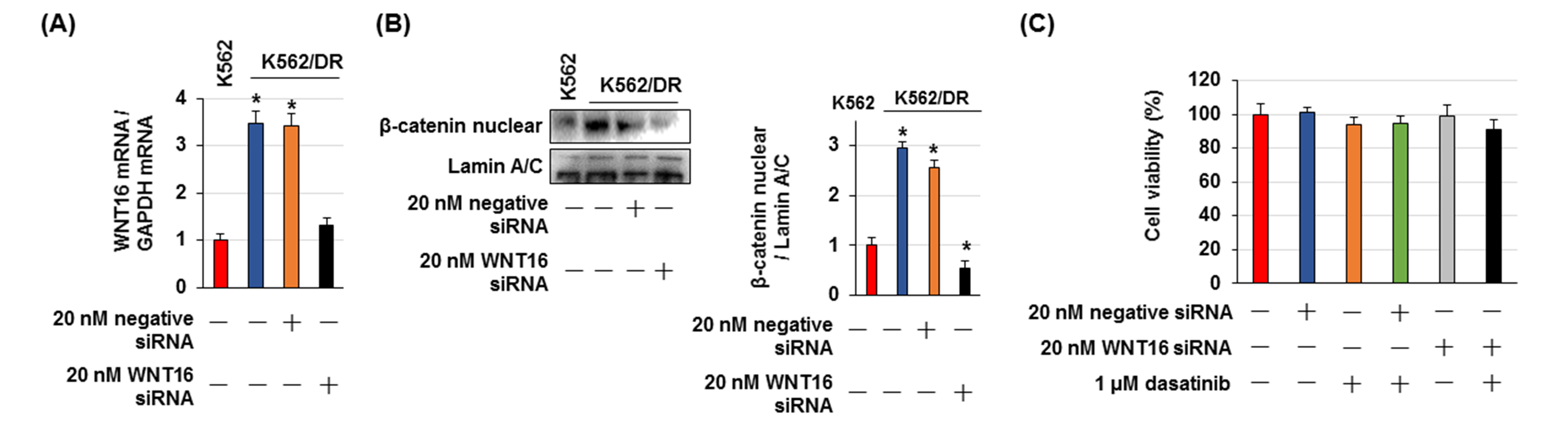

Supplement: Supplementary file 3 — Figure S2. Effect of WNT16 siRNA on dasatinib resistance of K562/DR cells. (A) K562/DR cells were administrated with siRNA of WNT16 or a negative control for 1 day, and RNA were extracted. WNT16 levels were examined by real time PCR. The results were standardized using GAPDH values and then expressed as a test: control ratio. These results are the average of five independent experiments. *p < 0.01 versus untreated K562 cells. (B) Cell lysates were analysed by western blotting. β‐catenin was analysed by densitometry and were standardized to Lamin A/C. (C) K562/DR cells were administrated with the demonstrated concentrations of WNT16 siRNA or dasatinib. After incubation for 72 h, the number of surviving/dead cells was determined by trypan blue staining. These results are the average of five independent experiments. *p < 0.01 versus untreated K562/DR cells. [file CPR-56-e13420-s005.tif]

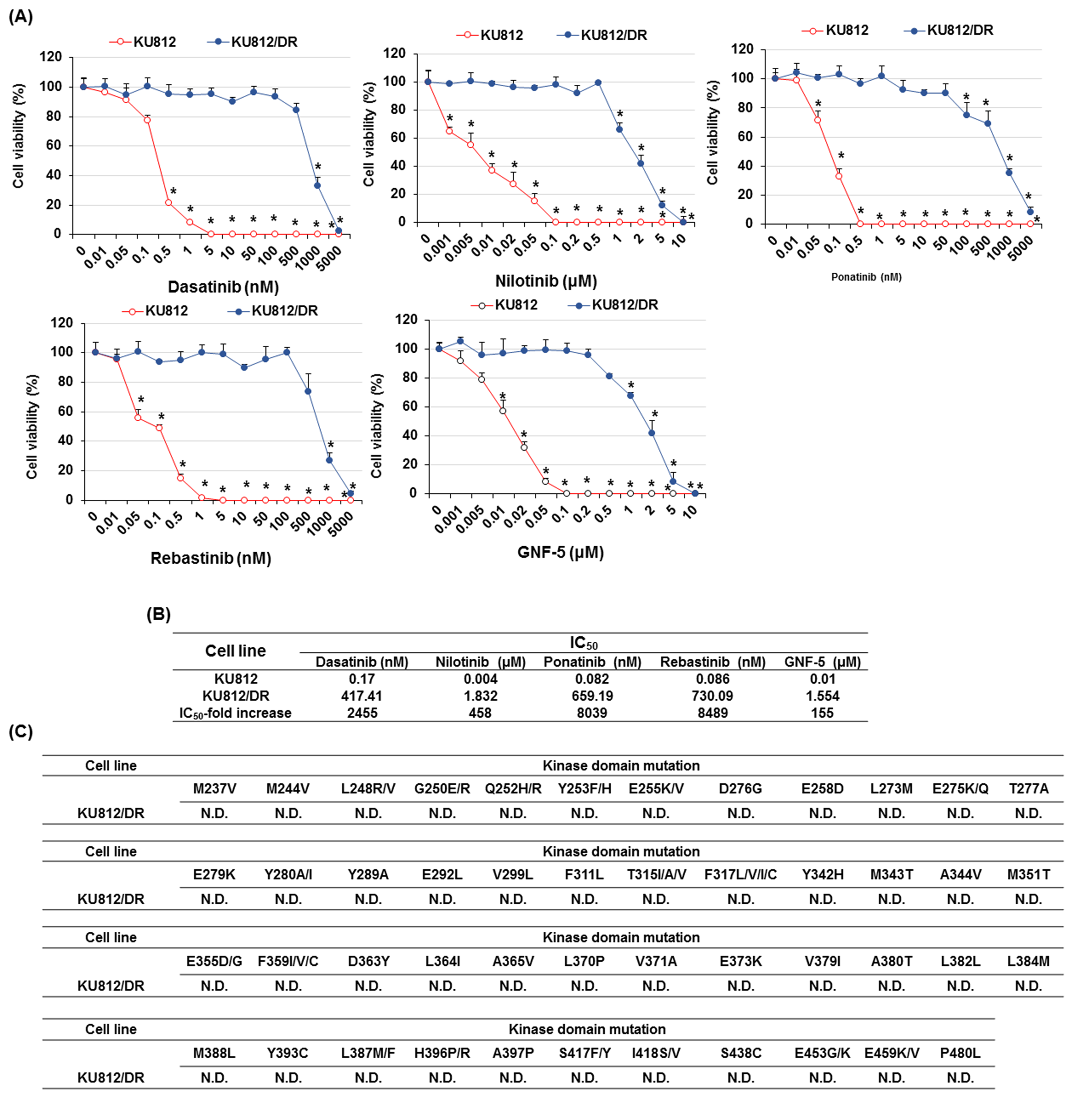

Supplement: Supplementary file 4 — Figure S3. Dasatinib and other BCR::ABL1 TKIs treatment did not increase cell death in KU812/DR cells. (A) Survival of KU812/DR and KU812 cells after treatment to various concentrations of dasatinib, nilotinib, ponatinib, rebastinib and GNF‐5 for 72 h; These results are the average of five independent experiments. *p < 0.01 versus untreated KU812 cells (evaluated by Dunnett's test). (B) The IC50 was evaluated by using a logistic curve for the data. (C) BCR::ABL1 mutations in KU812/DR cells were examined by NGS. [file CPR-56-e13420-s003.tif]

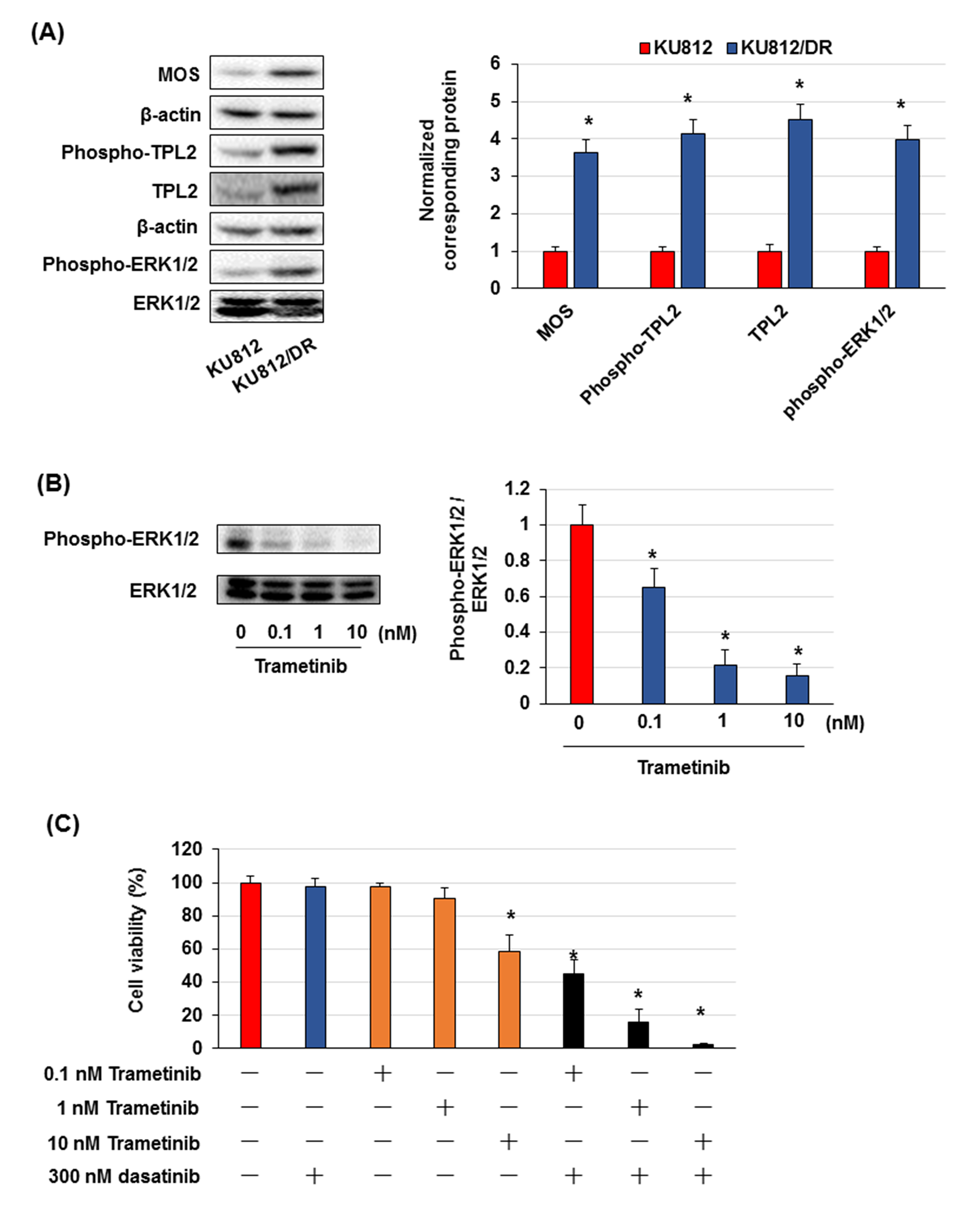

Supplement: Supplementary file 5 — Figure S4. Elevated expression of MOS, TPL2, and ERK1/2 contributed to dasatinib resistance in KU812/DR cells. (A) Cell lysates were analysed by western blotting. Proteins were analysed by densitometry and were standardized to β‐actin or ERK1/2. (B) KU812/DR cells were administrated with trametinib for 72 h. Cell lysates were analysed by western blotting. Phosphorylated ERK1/2 were analysed by densitometry and were standardized to ERK1/2. (C) KU812/DR cells were administrated with 0.1, 1, and 10 nM trametinib or 300 nM dasatinib. After incubation for 72 h, the number of surviving/dead cells was determined by trypan blue staining. These results are the average of five independent experiments. *p < 0.01 versus untreated KU812/DR cells (evaluated by Dunnett's test). [file CPR-56-e13420-s001.tif]

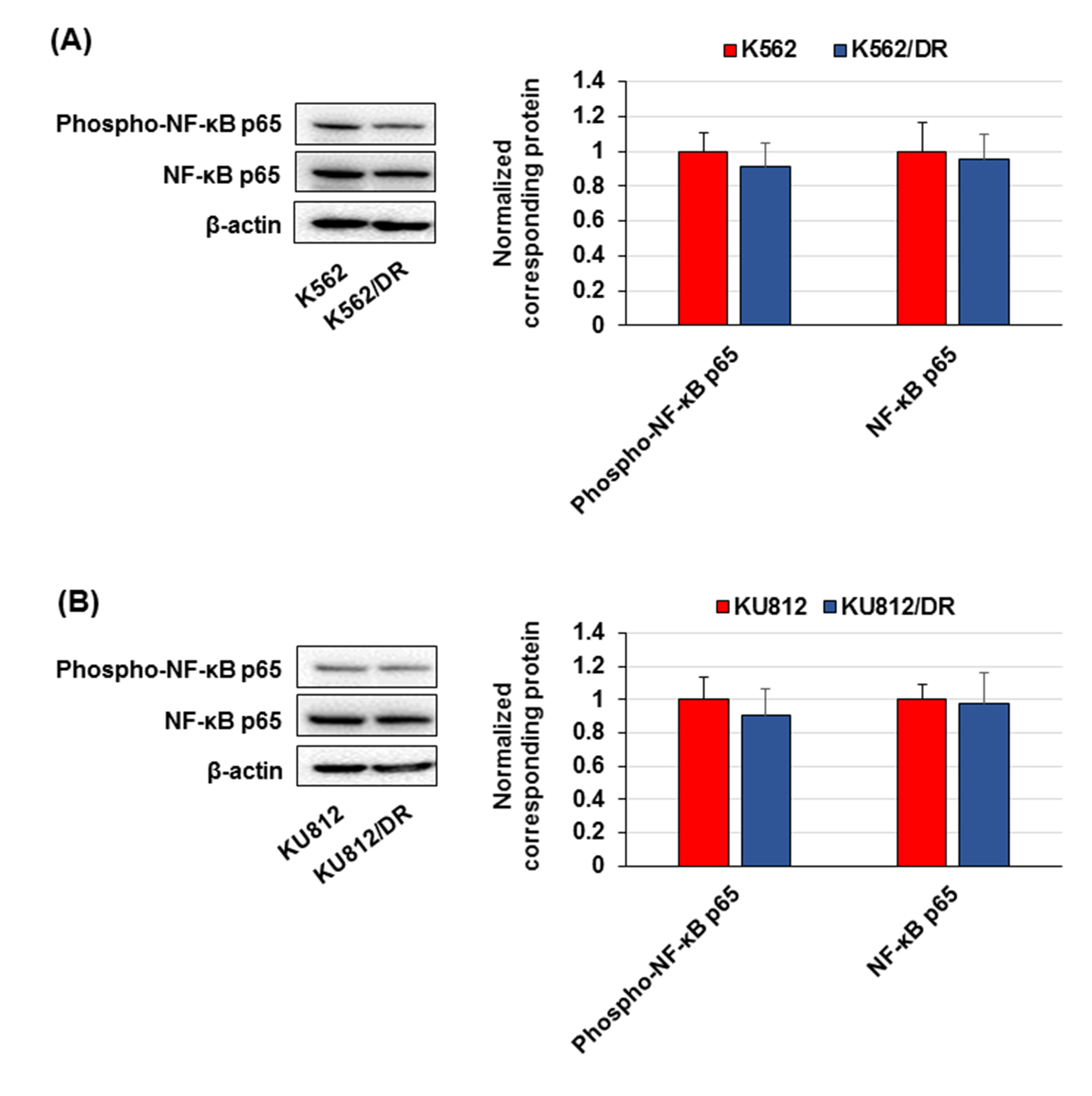

Supplement: Supplementary file 6 — Figure S5. Expression of phosphorylated and total NF‐κB p65 on K562, K562/DR, KU812 and KU812/DR cells. (A) K562 and K562/DR, (B) KU812 and KU812/DR cell lysates were analysed by western blotting. Proteins were analysed by densitometry and were standardized to β‐actin. [file CPR-56-e13420-s004.tif]
